# Resveratrol’s bibliometric and visual analysis from 2014 to 2023

**DOI:** 10.3389/fpls.2024.1423323

**Published:** 2024-10-08

**Authors:** Haoyue Wei, Guowei Fang, Weina Song, Hongye Cao, Ruizhe Dong, Yanqin Huang

**Affiliations:** ^1^ The First Clinical Medical College, Shandong University of Traditional Chinese Medicine, Jinan, China; ^2^ Department of Endocrinology, Affiliated Hospital of Shandong University of Traditional Chinese Medicine, Jinan, China; ^3^ Department of Pediatric Respiratory and Critical Care, Qilu Hospital of Shandong University Dezhou Hospital, Dezhou, China

**Keywords:** resveratrol, bibliometrics, Web of Science, VOSviewer, CiteSpace, pharmacological action

## Abstract

**Introduction:**

Resveratrol (RSV) is a natural polyphenolic compound derived from a variety of plants that possesses a wide range of biological activities, including antioxidant, anti-inflammatory, antitumor, antibacterial, antiviral, anti-aging, anti-radiation damage, anti-apoptosis, immune modulation, regulation of glucolipid metabolism, inhibition of lipid deposition, and anti-neuro. It is therefore considered a promising drug with the potential to treat a wide range of diseases.

**Method:**

In this study, using Web of Science Core Collection (WoSCC) and CiteSpace bibliometric tool, VOSviewer quantitatively visualized the number of countries, number of authors, number of institutions, number of publications, keywords, and references of 16,934 resveratrol-related papers from 2014–2023 for quantitative and qualitative analysis.

**Results:**

The results showed that an average of 1693.4 papers were published per year, with a general upward trend. China had the most publications with 5877. China Medical University was the institution with the largest number of publications and the highest number of citations in the field. The research team was mainly led by Prof. Richard Tristan, and the journal with the highest number of published papers was *Molecular. Dietary polyphenols, oxidative stress, and antioxidant and anti-inflammatory effects* are the most frequently cited articles. Oxidative stress, apoptosis, expression, and other keywords play an important role in connecting other branches of the field.

**Discussion:**

Our analysis indicates that the integration of nanoparticles with RSV is poised to become a significant trend. RSV markedly inhibits harmful bacteria, fosters the proliferation of beneficial bacteria, and enhances the diversity of the intestinal flora, thereby preventing intestinal flora dysbiosis. Additionally, RSV exhibits both antibacterial and antiviral properties. It also promotes osteogenesis and serves a neuroprotective function in models of Alzheimer's disease. The potential applications of RSV in medicine and healthcare are vast. A future research challenge lies in modifying its structure to develop RSV derivatives with superior biological activity and bioavailability. In the coming years, innovative pharmaceutical formulations of RSV, including oral, injectable, and topical preparations, may be developed to enhance its bioavailability and therapeutic efficacy.

## 1 Introduction

RSV is a natural polyphenol, an antitoxin produced by many plants when they are stimulated, also known as 3,5,4’-trihydroxystilbene, with a molecular formula of C14H12O3, a relative molecular weight of 228.25, and both cis and trans configurations. It belongs to the non-flavonoids class of polyphenolic compounds (Salehi et al., 2018). Mainly found in grapes, tiger nuts, peanuts, blueberries, mulberries, cassia seeds, and *Polygonum multiflorum* (Tian and Liu, 2020). Modern pharmacological studies have shown that RSV has a variety of pharmacological effects, including anti-inflammatory (Berman et al., 2017), antioxidant (Meng et al., 2020), and other pharmacological activities. With the deepening research on the pharmacological effects of RSV, it has been found that RSV exerts its pharmacological effects in the treatment of diabetes (Barber et al., 2022), obesity (Barber et al., 2022), antibacterial, anti-atherosclerosis (Chen et al., 2016), and neuroprotection (Azargoonjahromi and Abutalebian, 2024) through multiple pathways and multiple targets.

In addition to being published in the review section, many bibliometric articles are also published in the systematic review section (Gu et al., 2021; Zhu et al., 2022; Liu et al., 2024; Zhi et al., 2024). Bibliometrics is similar to systematic reviews in terms of searching the literature and can be used to study different and important areas of investigation and to obtain a general overview of the published literature (Lubowitz et al., 2023). Bibliometrics reviews all fully published articles in biomedical journals, including diverse research methodologies such as descriptive studies, observational studies, experimental studies, qualitative studies, and systematic reviews, thus illuminating the robustness of the available evidence (Manoj Kumar et al., 2023). The analysis of bibliometrics data is facilitated by specialized software such as VOSviewer and CiteSpace (Manoj Kumar et al., 2023). Given that bibliometrics often engages with extensive data sets from databases, it encompasses processes like data retrieval, preprocessing, network extraction, normalization, mapping, analysis, and visualization, with findings presented through tables, citation maps, web displays, and graphical representations. Bibliometrics furnishes a holistic perspective and analytical insights into research fields, essential for discerning research trends and impacts, particularly when systematic reviews necessitate substantial literature data for contextual evaluation. The interrelation between bibliometrics and systematic reviews lies in their shared objective of summarizing and analyzing existing research, albeit with distinct emphases. Bibliometrics prioritizes quantitative metrics such as reference counts, citation patterns, and research trajectories, while systematic reviews concentrate more on the quality of research and the appraisal of results. Though bibliometrics may not directly belong to the realm of bioinformatics, the insights and analyses it offers can furnish valuable contextual information, trend perspectives, and resource evaluations pertinent to bioinformatics research (Montazeri et al., 2023). While bibliometrics does not directly assess research quality, systematic reviews may utilize this data to pinpoint and evaluate high-impact, widely cited studies. Furthermore, bibliometrics can underscore recent advancements and pressing issues within a discipline, thereby guiding systematic reviews to concentrate on contemporary hot topics and principal challenges.

In the past few years, there have been a lot of research papers on RSV. These papers summarize the role and mechanism of RSV, look at the current state of research, make suggestions for future research, and give a scientific basis for its development and use. Previous bibliometric analyses have elucidated the pivotal role and mechanisms of RSV in anticancer research. The emerging frontiers in this field are anticipated to concentrate on topics such as resveratrol-induced apoptosis, tumor microenvironment, and synergistic effects (Fu et al., 2024). Another bibliometric study explored RSV’s impact on cognitive function, assessing its influence on various cognitive disorders and underlying mechanisms. Areas of focus include the regulatory effects of RSV on neuronal damage in healthy adults, older adults, postmenopausal women, Alzheimer’s disease patients, individuals with diabetes-related cognitive impairments, psychiatric disorders, post-stroke cognitive deficits, and neonatal ischemic injury (Tu et al., 2023). A study on RSV and neuroinflammation suggests that oxidative stress and inflammatory responses are critical in neurodegenerative diseases, and RSV may mitigate these processes through its antioxidant and anti-inflammatory properties (dos Santos et al., 2024). RSV exerts significant anti-inflammatory effects, mitigating HIF-1α-mediated angiogenesis and impeding the progression of Asperger Syndrome through the TLR4/NF-κB signaling pathway (Guo et al., 2023). In 2022, Robin Haunschild and Werner Marx reviewed 3,344 publications on the health benefits of RSV in wine and grapes. Their findings indicate that moderate red wine consumption may offer health advantages, whereas excessive consumption could lead to cirrhosis and cancer. The study categorized the authors’ keywords into six domains: beverage-related, compound-related, disease-related, effect-related, mechanism-related, and broader keywords, and analyzed their usage (Haunschild and Marx, 2022).

Previously, Andy Wai Kan Yeung and colleagues conducted bibliometric research on countries contributing significantly to RSV research, highlighting major publications and their focal areas. Over half of the pertinent literature has emerged since 2013, partly due to heightened scientific activity in China. This review provides a succinct overview of research on RSV and its derivatives (Yeung et al., 2019). Our research conducts a bibliometric and visual analysis of RSV-related literature spanning from 2014 to 2023. It provides a thorough examination of various aspects, including research countries, institutions involved, author collaborations, journal publications, highly cited works, and keywords. Building upon previous bibliometric studies, this paper explores the comprehensive effects and mechanisms of RSV concerning cancer prevention, cognitive function, and neuroinflammation. Currently, our understanding of the specific mechanisms underlying RSV’s effects remains insufficient, necessitating further foundational research. Future investigations should emphasize the efficacy and safety of RSV in practical clinical settings.

## 2 Materials and methods

### 2.1 Data sources

We selected the WoSCC as the primary database for this study because of its extensive coverage of over 12,000 scholarly journals and frequent use by researchers. We used “Resveratrol” as the search term, set the search period as January 2014–December 2023, set the language as “English,” and limited the types of articles to “article” and “review article.” The search deadline was March 18, 2024. We excluded conference proceedings, letters, online publications, retractions, and other irrelevant documents, resulting in a total of 17,434 RSV-related documents. Two researchers independently searched and screened the above data, with the third senior researcher making the final decision in cases of disagreement. We imported the data into CiteSpace to remove duplicates, and we finally obtained 16934 articles, including 2772 reviews and 14162 articles. We conducted all literature searches and downloads on the same day to reduce the number of citations resulting from frequent database updates.

### 2.2 Bibliometric analysis software

**Figure 1:**
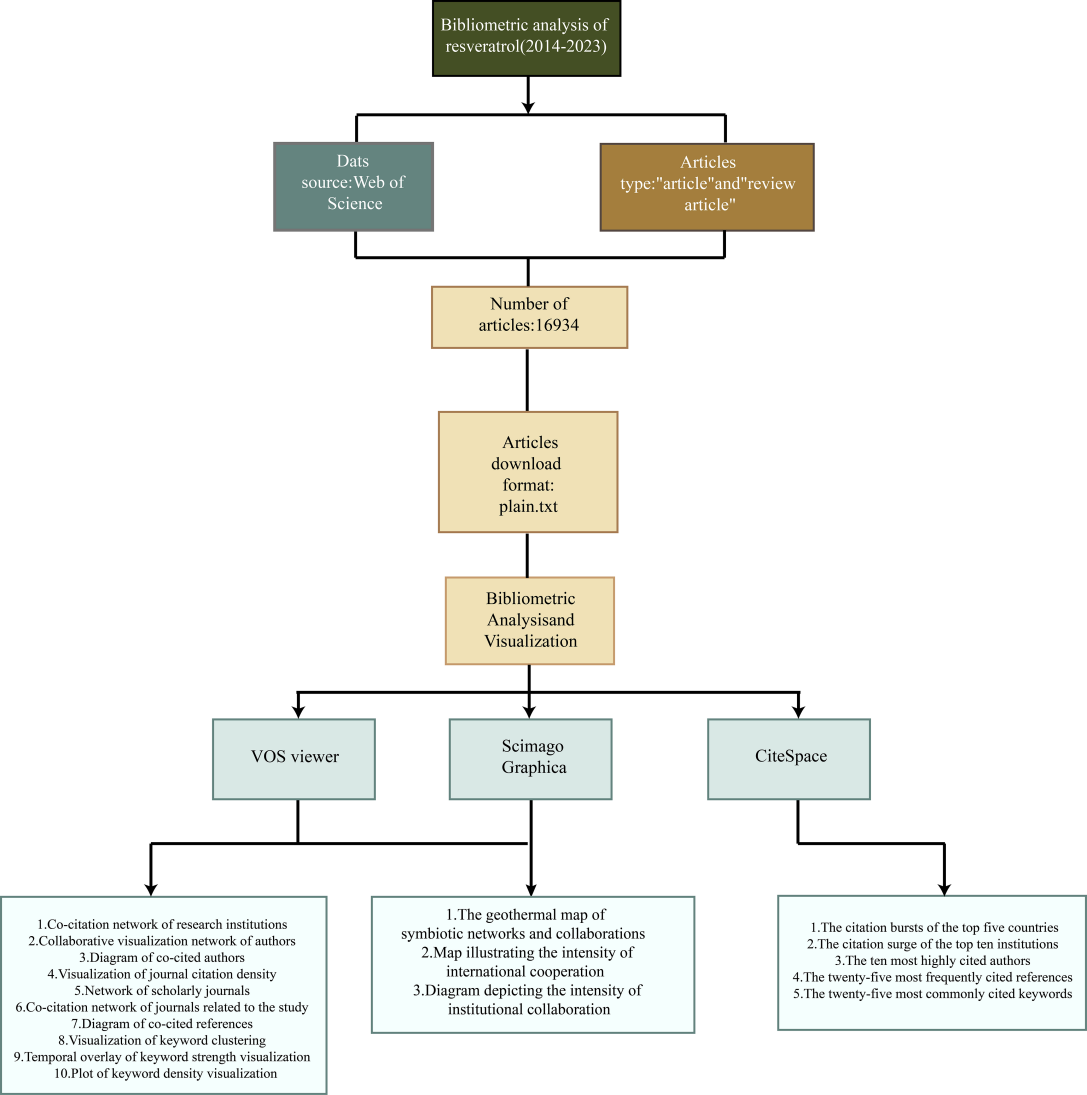
Flowchart of CiteSpace bibliometric analysis.

We exported the screened documents in plain text and tab-delimited file formats, containing full records and cited references, respectively, and then imported them into VOSviewer 1.6.18, CiteSpace 6.1.6, Pajek 5.16, Scimago Graphica 1.0.35, and the “Bibliometrix” R software package (R Studio, version 4.2.0). “Bibliometrix” R package (R Studio, version 4.2.0). The main indicators analyzed were the number of publications, country/institution/author partnerships, keyword emergence and co-occurrence clustering, and trends in research themes (**Figure 1**).

## 3 Results and analysis

### 3.1 RSV literature volume and trend analysis

**Figure 2:**
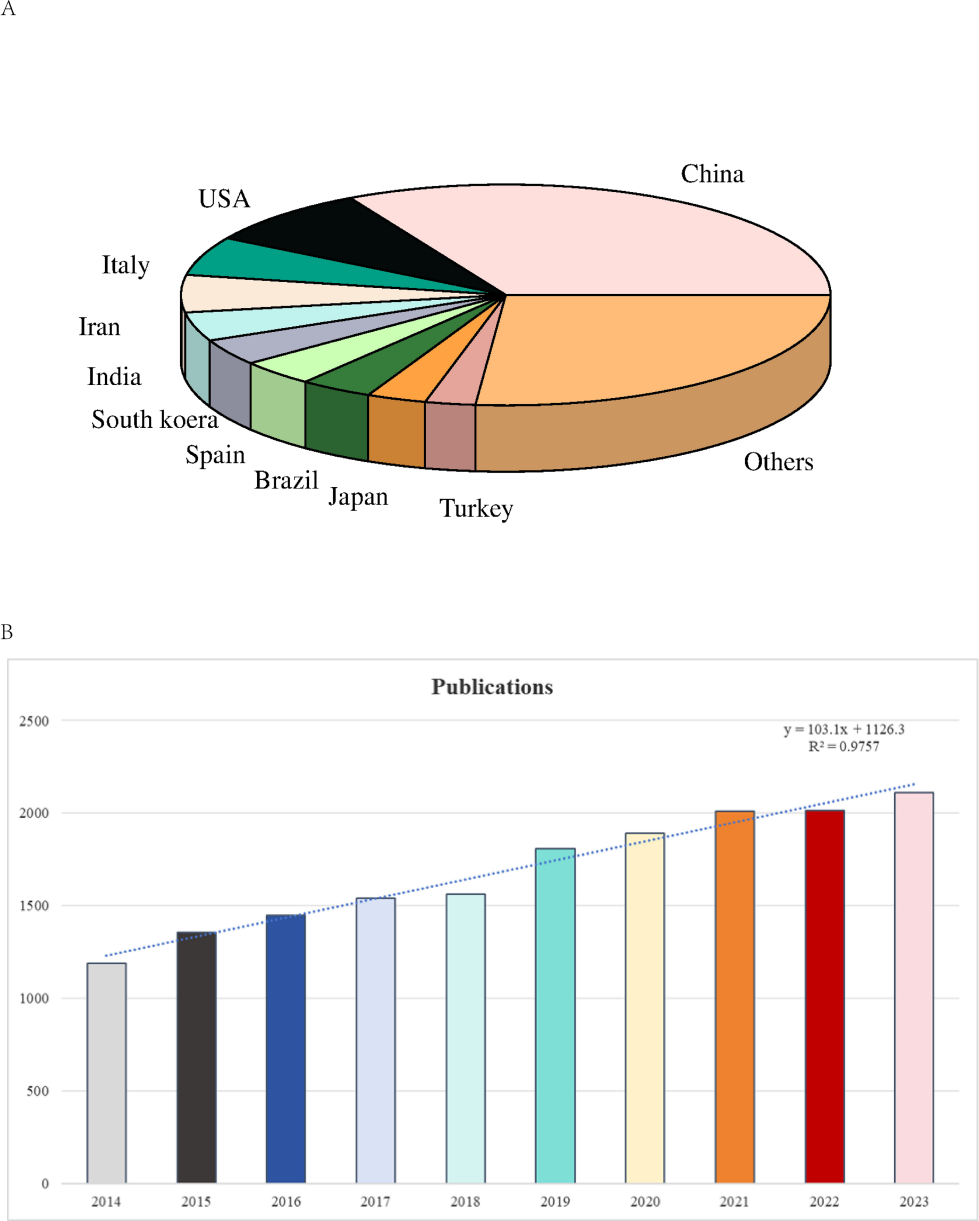
**(A)** Pie chart of RSV research publications by country. **(B)** Research publications on “RSV” from 2014 to 2023 (increasing trend).

In this pie chart, we illustrate the distribution of publications across various countries in the domain of RSV research (**Figure 2A**). The chart reveals that China holds a predominant and unequivocal lead in the volume of published works, whereas other nations such as the United States, Italy, and Iran contribute a relatively modest share. This data underscores that China’s research efforts in RSV are significantly more concentrated compared to those of other countries. The color-coded markers in the chart facilitate a clear identification of the publication percentages by country. The volume of publications within a specified timeframe reflects the trajectory of research endeavors. From 2014 to 2023, we compiled a total of 16,934 papers (comprising 2,772 review articles and 14,162 research articles) about RSV, averaging 1,693.4 publications annually. Over the past decade, there has been a discernible and steady rise in academic output in this field. Since 2017, annual publications have consistently surpassed 1,500, culminating in a peak of 2,112 publications in 2023. This represents a 77.48% increase compared to 2014, underscoring the substantial research value of this area and a notable expansion in research activity. Furthermore, there has been a marked upsurge in the exploration of RSVs. To illustrate the annual publication trend, we employed the exponential equation (y = 103.1x + 1126.3) (R² = 0.9757), where (x) denotes the year and (y) signifies the annual publication volume. The fitted curve accurately represents this equation (**Figure 2B**), predicting a sustained upward trend in annual publications, reflecting an escalating interest in RSVs. Thus, it is plausible to anticipate a period of significant advancement in this domain in the forthcoming years. The investigation into RSV has garnered substantial scholarly interest, as evidenced by the rising number of academic publications. This emerging field is attracting more researchers and emphasizing its importance in the academic community. We anticipate the field’s continued expansion and the innovative role of novel nanomedicines in enhancing the therapeutic efficacy of RSV. This growth is expected to attract additional funding, talent, and stakeholder engagement, fostering heightened investment and collaboration between academia and industry in RSV-related innovations and technologies.

### 3.2 Distribution of RSV research countries

**Figure 3:**
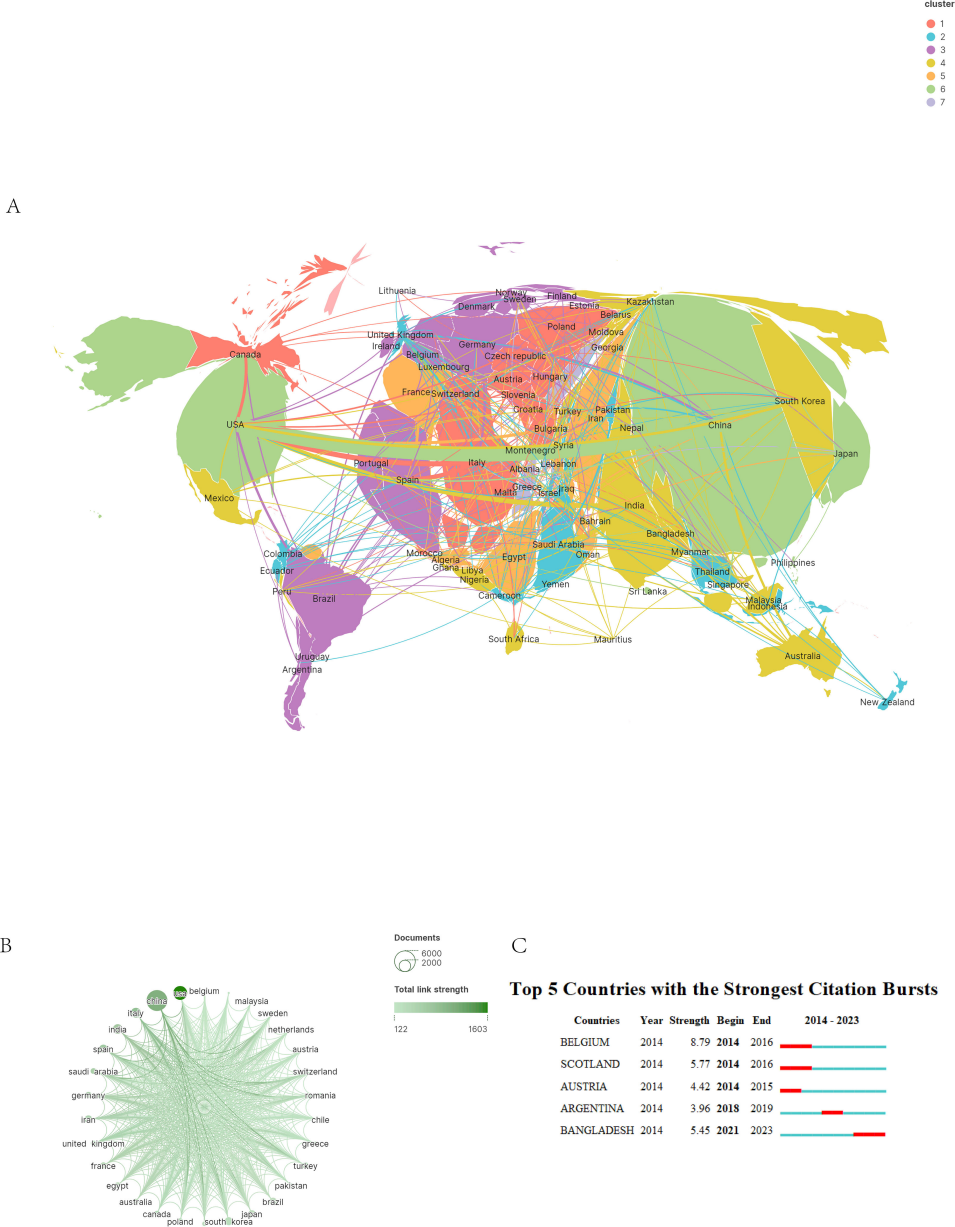
**(A)** Shows the geothermal map of symbiotic networks and cooperation. The contours of each country’s territory differentiate it, while the thickness of the lines connecting different countries indicates the intensity of their cooperation. In addition, the color of the different countries and the size of the circles represent the number of publications. **(B)** Map of the intensity of country cooperation. **(C)** The map displays the citation bursts of the top 5 countries, with each country’s bursts represented by red bars.

A total of 136 countries and regions have published RSV articles by scholars, and we use the “Bibliometrix” R package to visualize the network of national collaborations. The cooperation between these regions forms four different clusters. The thickness of the lines connecting the spheres indicates the degree of cooperation between countries. Each sphere represents a country. The size of the sphere represents the total number of articles published by the country in the time period, and the color of the sphere represents the intensity of cooperation between the country and other countries. According to the global productivity map (**Figure 3A**), most of the papers are published in Asian, North American, and European countries. China published the largest number of papers, with 5,877, or 34.7% of the total, followed by the United States with 14.2% (n = 2404) and Italy with 7.04% (n = 1192) (**Table 1**). This is closely related to the level of economic development in these countries and the importance attached to scientific research by their respective governments. The number of publications often reflects a country’s or region’s status in the field. China and the United States have the most publications in RSV, indicating their academic status and influence. Close cooperation between the two countries will promote theoretical innovation and address existing technical challenges in the field.

**Table 1:** Top 10 countries in the WoSCC of research publications in the RSV field.

Number	Country	Number of publications	Number of citations	Average number of citations per article
**1**	China	5419	122969	22.69
**2**	USA	2404	82379	34.27
**3**	Italy	1192	34077	28.59
**4**	India	1149	26972	23.47
**5**	Spain	855	24772	28.97
**6**	South Korea	789	19893	25.21
**7**	Iran	753	18513	24.59
**8**	Brazil	724	13692	18.91
**9**	Japan	618	11666	18.88
**10**	Turkey	506	8416	16.63

This graph illustrates the strength of international collaboration in RSV research (**Figure 3B**). Each node represents a country, with its size proportional to the total number of publications in RSV research from that nation. The color gradient in the figure denotes the intensity of cooperation, with lighter hues indicating a lower frequency of collaboration and darker hues signifying a higher frequency. For instance, the thicker connections between China and the United States, South Korea, Poland, and Turkey highlight a robust network of frequent cooperation. This visualization reveals that the United States, China, and several European and Asian countries are central to global RSV research collaborations, depicting a comprehensive international cooperation landscape.

Citation bursts are critical for identifying projects with a significant increase in the number of citations within a given timeframe. They provide insights into the dynamics and trajectory of a research field. Analyzing projects with rapid citation growth can help researchers identify emerging trends in a given research area that have caught their attention. We display the citation explosion for the top 5 countries (**Figure 3C**). From 2014 to 2016, Belgium and Scotland showed a significant increase in the number of publications, as indicated by the dark red line in the figure. This line reflects the intensity of the citation explosion in the most influential countries. Notably, Bangladesh, a developing nation in Asia, has demonstrated a comparable trend in publication numbers from 2021 to 2023, similar to the established European countries. This observation suggests that developed nations in Europe and America are no longer the sole contributors to RSV research, as developing countries are increasingly dedicating resources to this field. These results underscore the necessity for global collaboration, especially to harness diverse knowledge and expertise from various regions. Monitoring emerging trends such as citation bursts is crucial for remaining informed about evolving areas of interest and innovation. Additionally, these findings serve as a call to industry practitioners to consider global partnerships for exploring investment opportunities or expanding markets. Broadening research across geographic boundaries enhances both expertise and innovative potential. The global distribution of RSV research, patterns of collaboration, and emerging trends signify a shift towards more inclusive global engagement. These findings emphasize the urgency of broader collaborations, highlight emerging research hubs, and suggest opportunities for researchers and industry stakeholders to leverage diverse expertise in addressing RSV.

### 3.3 Institutional distribution of RSV research areas

**Figure 4:**
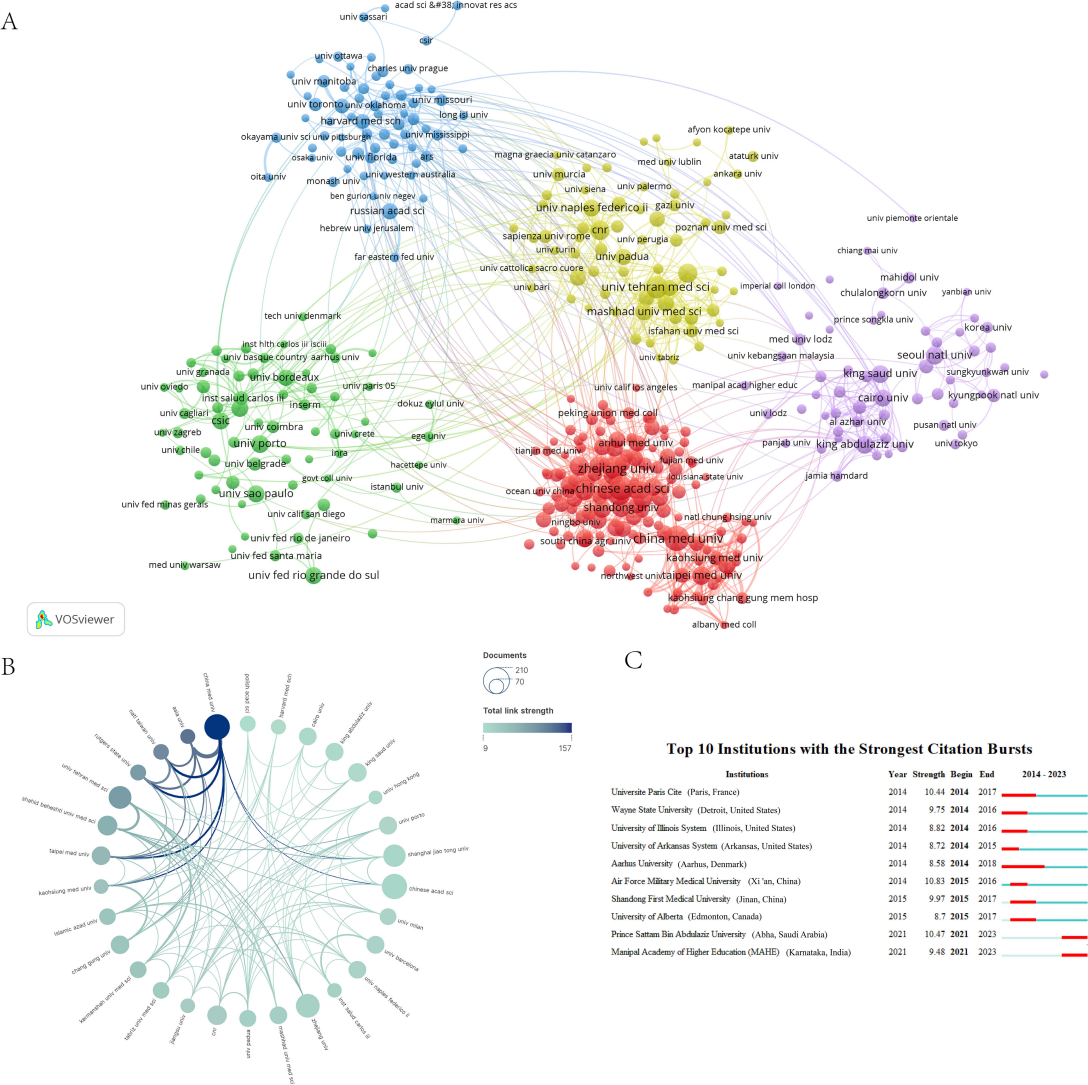
**(A)** Shows a co-citation network of related research institutions. Different colors indicate different clusters, and institutions with strong co-citation relationships are clustered to generate a hierarchical graph describing these associations. The thickness of the lines between the circles indicates the strength of cooperation between institutions, while the size of the circles represents the number of documents published by each institution. **(B)** Graph of the intensity of cooperation between institutions. **(C)** The citation explosion of the top 10 institutions is depicted, with the red bars representing the period during which the institutions experienced this explosion.

Between 2014 and 2023, a total of 400 organizations will participate in RSV-related research. China Medical University (Shenyang, China), Chinese Academy of Sciences (Beijing China), Zhejiang University (Hangzhou China), Tehran Medical University (Tehran, Iran), Shanghai Jiao Tong University (Shanghai China), Nanjing Medical University (Nanjing, China), Jiangnan University (Jinan, China), Shahid Beheshti University of Medical Sciences (Tehran, Iran), Consiglio Nazionale delle Ricerche (Catania, Italy), and Fudan University (Shanghai China) are among the top ten research organizations in terms of the number of publications (**Table 2**). 7 of them belong to China, 2 to Iran, and 1 to Italy. China Medical University (Shenyang, China) has the largest number of publications and the most citations in this field, indicating that it is in the leading position in RSV research. It is worth noting that Harvard University (Cambridge, United States) has the highest average number of citations (89.41), indicating that its research results are widely recognized by the academic community, but it is not among the top 10 institutions in terms of the number of publications. **Figure 4A** illustrates the co-citation network of research institutions focused on RSV. We constructed the network of research institutions’ cooperation relationship with the threshold of ≥5 publications and divided the network into 5 clusters according to color, the width of the connecting line represents the intensity of cooperation between institutions, and the size of the circle represents the number of institutional publications. Tehran Medical University (Tehran, Iran) and Consiglio Nazionale delle Ricerche (Rome, Italy) serve as the central institutions within the yellow cluster, exhibiting numerous co-citations. Harvard University (Cambridge, United States) leads the blue cluster in terms of the volume of published papers and co-citations. Chinese universities in the red cluster are notable for their substantial publication output and strong co-citation ties, establishing a core group with significant collaboration potential. King Saud University (Riyadh, Saudi Arabia) and King Abdulaziz University (Jeddah, Saudi Arabia) are pivotal institutions in the purple cluster, characterized by numerous shared citations. Core institutions of the green cluster include Universidade Federal do Rio Grande do Sul (Porto Alegre, Brazil) and Universidade do Porto (Porto, Portugal). Through an analysis of this co-citation network, we can discern key institutions and collaborative networks in RSV research, observe regional centralization trends among different clusters, and uncover potential opportunities for collaboration. In terms of inter-institutional cooperation, China Medical University, as a leading institution, shows a strong willingness to cooperate with other institutions. Strong links between China Medical University (Shenyang, China) and almost all prestigious academic organizations support this (**Figure 4B**). It is noteworthy that the vast majority of these institutions show a preference for national rather than international collaboration. Universite Paris Cite (Paris, France), Wayne State University (Detroit, United States), and the University of Illinois System (Illinois, United States) are the top three institutions in terms of citation bursts, unfortunately, none of the three institutions have shown any sustained bursts in the previous five years (**Figure 4C**).

**Table 2:** Top 10 organizations in the WoSCC of RSV research publications.

Number	Institution	Country	Publications	Citations	Average number of citations per article
**1**	China Medical University	China	185	4373	23.64
**2**	Chinese Academy of Sciences	China	181	4268	23.58
**3**	Zhejiang University	China	159	3711	23.34
**4**	Tehran University of Medical Sciences	Iran	147	3826	26.03
**5**	Shanghai Jiao Tong University	China	142	4144	29.18
**6**	Nanjing Medical University	China	114	2878	25.25
**7**	Jiangnan University	China	112	3250	29.02
**8**	Shahid Beheshti University of Medical Sciences	Iran	111	3636	32.76
**9**	Consiglio Nazionale delle Ricerche	Italy	108	3105	28.75
**10**	Fudan University	China	108	3444	31.89

### 3.4 Analysis of research author collaborations in the field of RSV

**Figure 5:**
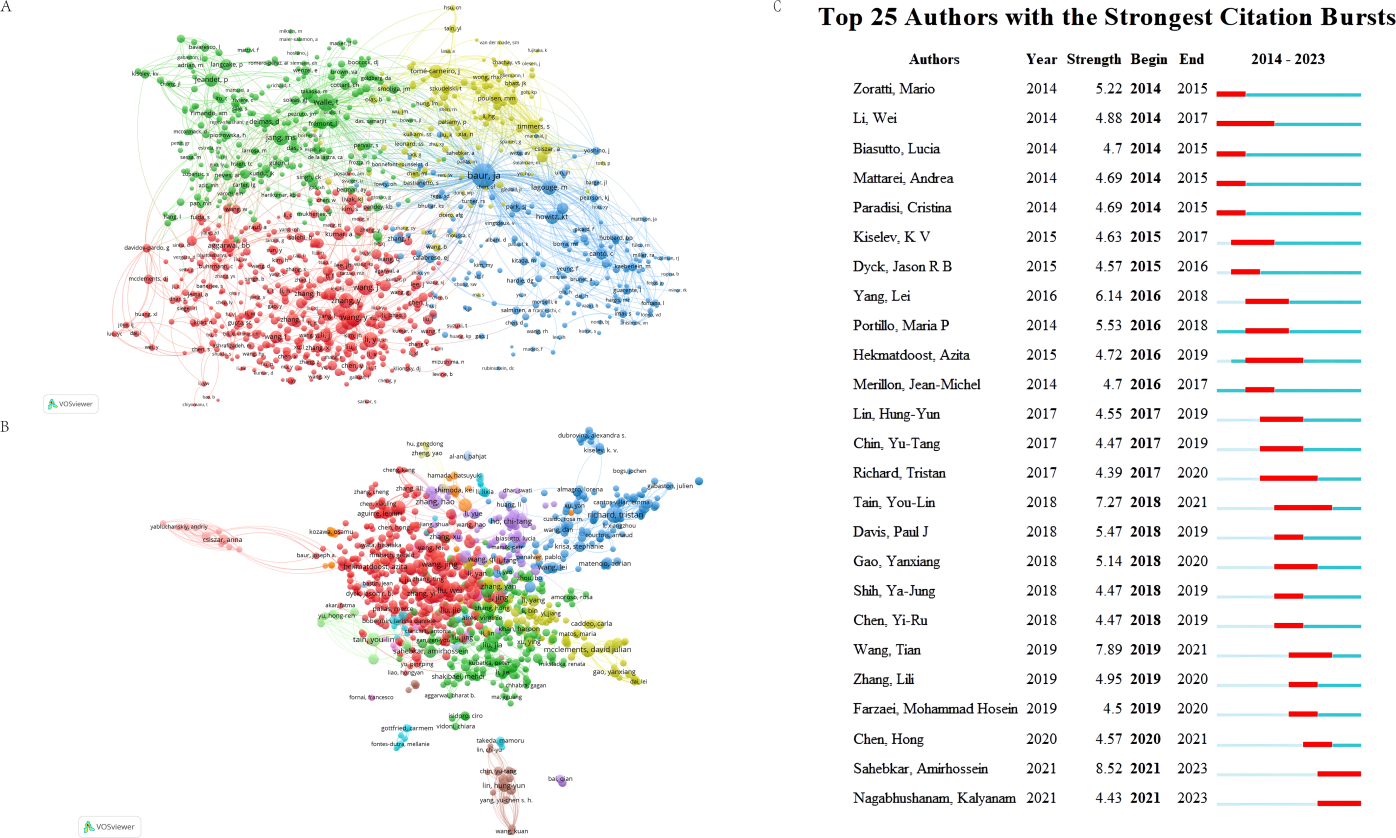
**(A)** Shows the collaborative visualization network of authors in the RSV research area. Circles and text labels form a node where different colors indicate different clusters. The size of the circles positively correlates with the number of articles published by the authors, while the thickness of the lines between the circles indicates the intensity of collaboration between the authors. **(B)** Graph of co-cited authors. The circle’s size signifies the number of times an author has received co-citations, while the line connecting the circles signifies the co-citation relationship within the literature. **(C)** The top 10 authors with the highest number of citations in RSV-related publications.

Highly published and highly cited authors have a very high contribution and influence in the field of RSV, which can provide benchmarks and guidance for future research and help other researchers explore emerging research areas and potential innovations. According to Price’s law, the formula N=0.749×(ηmax)1/2(ηmax indicates the amount of literature of the author with the largest number of publications) was used to determine the core authors, and when the number of publications of an author>N, the author is the core author. Following the aforementioned screening criteria, we identified 1354 core authors who authored a total of 12933 articles, representing 76.37% of the total article count. This suggests that these core authors have made a significant contribution to the majority of the RSV research literature. In addition, Richard Tristan (Université de Bordeaux, France), Zhang Hao (College of Animal Science and Technology, China), and Liu Wei (Huazhong University of Science and Technology, China) are the three authors with the largest number of publications, with 51, 42, and 41 articles (**Table 3**). These authors’ contributions emphasize their significance in the field of RSV. We identified the core authors as those with more than 6 publications, visualized and analyzed the collaborations among 1000 authors with more than 10 publications, and displayed the authors’ collaborative network (**Figure 5A**). These authors have formed several stable collaborative structures, and there is close cooperation among the internal staff. However, there is a lack of exchanges and cooperation among the different groups, and a few groups have not established cooperation with other groups. We should aim to increase inter-regional exchanges and cooperation among the researchers in the future. In the future, we should focus on increasing cross-regional communication and cooperation among researchers. The citation burst displays the frequency of an author’s citations in a specific field over a specific timeframe. We show the top 10 authors with the highest citation frequency in their field (**Figure 5B**).

**Table 3:** Top 10 authors in terms of the number of publications and citations.

Number	Author	Institution	Country	Publications	Citations	Average number of citations per article
**1**	Richard tristan	Université de Bordeaux	France	51	1126	22.08
**2**	Zhang hao	College of Animal Science and Technology	China	42	828	19.71
**3**	Liu wei	Huazhong University of Science and Technology	China	41	971	23.68
**4**	Mcclements david julian	University of Massachusetts	USA	41	3359	81.93
**5**	Ho chi-tang	Rutgers University	USA	40	1003	25.08
**6**	Portillo maria p.	University of the Basque Country	Spain	38	931	24.50
**7**	Wan jing	The Third Military Medical University	China	38	904	23.79
**8**	Tain you-lin	Kaohsiung Chang Gung Memorial Hospital and Chang Gung University College of Medicine	China	35	883	25.23
**9**	Wang yan	Capital Medical University	China	35	876	25.03
**10**	Hamada hiroki	Okayama University of Science	Japan	34	283	8.32

Sahebkar Amirhossein (Mashhad University of Medical Sciences, Iran) has the highest citation burst (8.52), followed by Wang Tian (Nanjing Agricultural University, China) (citation burst = 7.89). In addition, we noticed that authors Chen Hong (Medical College of Chinese People’s Armed Police Forces, China), Sahebkar Amirhossein (Mashhad University of Medical Sciences, Iran), and Nagabhushanam Kalyanam (Sabinsa Corporation, USA) have significantly increased the number of publications in the last three years, which indicates that they are paying more attention to the field of RSV and may produce more research results in this field in the future (**Figure 5C**). Sahebkar Amirhossein’s research contribution to RSV is to provide its potential application in a variety of disease models, including diabetic nephropathy (Ghavidel et al., 2024), neurodegenerative diseases (Keshavarz Shahbaz et al., 2024), Parkinson’s disease (Iranshahy et al., 2022), and metabolic disorders (Ghavidel et al., 2023). These studies have laid the foundation for the clinical application of RSV and the development of novel treatment options.

### 3.5 A study of journal publications in the field of RSV

We used the bibliometrics online analysis platform to select journals with high publication volume and high impact in the field of RSV, and visualized the journals and co-cited journals through VOSviewer programming. We show the top 10 journals in terms of publications and co-citations, and their corresponding IF (JCR2024) and JCR numbers (**Table 4**). JCR Q1 and Q2 distribute the top 10 journals, with seven of them having IF values higher than 5. The number of publications in *Molecules* (MDPI, Switzerland) and *International journal of molecular sciences* (MDPI, Switzerland) is more than 400, indicating that these two journals are more active than others in the field. The highest number of publications was found in *Molecules* (IF = 4.6, Q2) with 440 papers, followed by *International journal of molecular sciences* (IF = 5.6, Q1) (429 papers) and *Nutrients* (MDPI, Switzerland) (IF = 5.9, Q1) (304 papers). *Molecules* is an academic journal of chemistry-organic chemistry, focusing on the frontiers of materials chemistry.

**Table 4:** Top 10 journals with RSV research publications in WoSCC.

Number	Journals	IF (2021)	IF (2022)	IF (2023)	JCR (2023)	Publisher	Countries	Count	Total citation	Average number of citations per article
**1**	Molecules	4.93	4.6	4.2	Q2	MDPI	Switzerland	440	8701	19.78
**2**	International journal of molecular sciences	6.21	5.6	4.9	Q1	MDPI	Switzerland	429	9537	22.23
**3**	Nutrients	6.71	5.9	4.8	Q1	MDPI	Switzerland	304	8623	28.37
**4**	Food chemistry	9.23	8.8	8.5	Q1	ELSEVIER	the United Kingdom	231	7755	33.57
**5**	Scientific reports	4.38	4.6	3.8	Q2	NPG	the United Kingdom	216	6750	31.25
**6**	Antioxidant	7.675	7	6.0	Q1	MDPI	Switzerland	208	3146	15.13
**7**	Plos one	3.752	3.7	2.9	Q2	PLOS	United States	205	7243	35.33
**8**	Journal of agricultural and food chemistry	5.895	6.1	5.7	Q1	ACS	United States	172	4668	27.14
**9**	Frontiers in pharmacology	5.988	5.6	4.4	Q1	Frontiers	Switzerland	167	3266	19.56
**10**	Food & function	6.317	6.1	5.1	Q1	The Royal Society of Chemistry	the United Kingdom	158	5723	36.22

**Figure 6:**
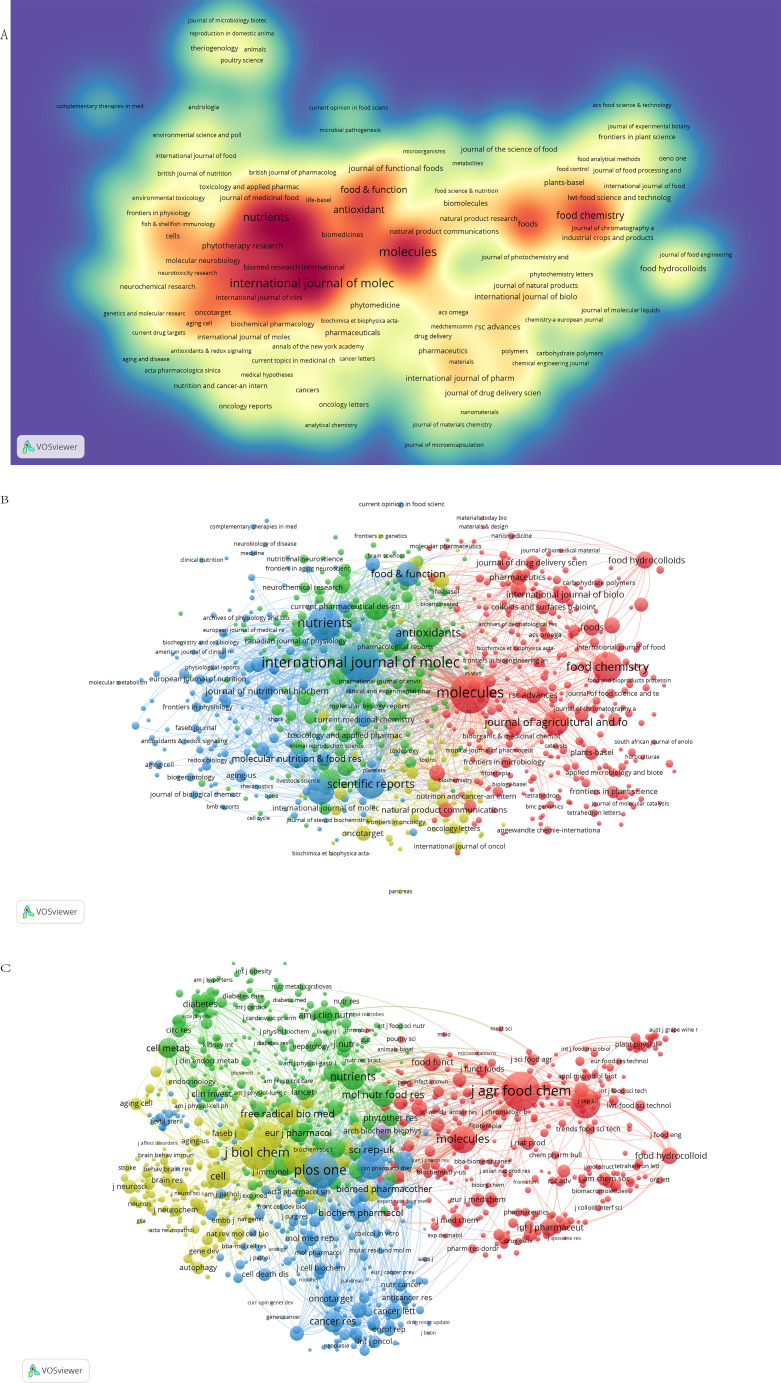
**(A)** Visualization of journal citation density. The color intensity represents the total number of journal publications, with the more reddish color bias representing a higher number of publications. **(B)** RSV network of research-related journals. Each node represents a journal. The same color indicates that the nodes are in the same cluster. Node size and link thickness reflect the frequency of journal collaboration. **(C)** RSV study-related journals co-citation network. the same color indicates that the nodes are in the same cluster. The node size reflects the frequency of journal co-citation, and the links represent the co-citation relationship between journals, proportional to the thickness of the links.

The visualization of journal citation density illustrates the distribution of journals within a two-dimensional framework, reflecting their citation impact (**Figure 6A**). Varied colors denote distinct citation densities, with darker hues typically indicating higher densities. Journals such as *International journal of molecular sciences*, *Molecules*, and *Nutrients* exhibit the highest citation densities. In addition, we used cluster analysis to roughly categorize all journals into four groups (**Figure 6B**). The entire network of journals is very closely and widely connected. The red area includes *journal of agricultural and food chemistry* (ACS, USA), *Molecules*, *international journal of pharmaceutics* (ELSEVIER, UK) and so on. The yellow area contains *Oncotarget* (Impact Journals), *Nutrition and Cancer an international journal* (Taylor & Francis). The green cluster contains *antioxidant* (MDPI, Switzerland), *molecular nutrition & food research* (Wiley). *Nutrients*, which ranks first in the number of scientific reports, belongs to the blue cluster. *Molecules*, which has the most average citations per article, belongs to the red cluster, and the *International journal of molecular sciences*, which ranks first in the number of citations, belongs to the blue cluster.

By analyzing the clusters (**Figure 6C**), we can directly observe the cooperation between journals and other information. The *journals of agricultural and Food chemistry*, *journal of biological chemistry*, *plos one*, and *Nutrients* (MDPI, Switzerland) are active journals with red, yellow, blue, and green clusters respectively, which have extensive co-citation relationships.

### 3.6 Analysis of highly cited literature in the field of RSV research

**Figure 7:**
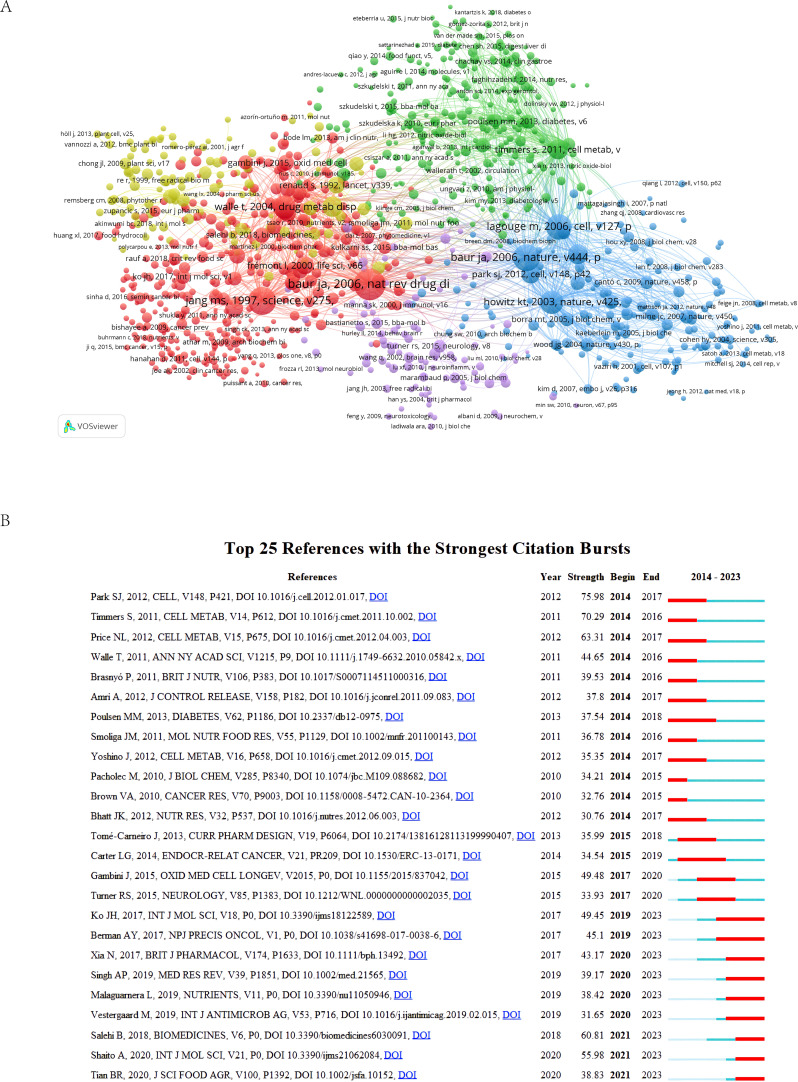
**(A)** Graph of co-cited references. The size of the spheres represents the co-citation frequency of articles in the RSV domain, and the connecting lines between the circles indicate the co-citation relationships in the literature. **(B)** The 25 most frequently cited references. The red line represents the burst interval, indicating the start and end years of the citation explosion, as well as the burst duration. The blue timeline represents the publication timeline, while the light and dark blue parts indicate the absence and presence of citations, respectively.

In the co-cited literature network within the domain of RSV research (**Figure 7A**), each cluster signifies a significant research topic or direction, facilitating the identification of influential studies. For instance, 2006 Joseph A Baur et al. published *Therapeutic Potential of Resveratrol: The In Vivo Evidence* and *Resveratrol Improves Health and Survival of Mice on a High-Calorie Diet*. This network reveals an extensive number of references and connections between nodes, underscoring a robust interrelationship with other scholarly works.

VOSviewer was used to look at RSV-related literature published from 2014 to 2023 (**Table 5**), and find important papers in the field by looking at literature co-citations and citation bursts, which show how research topics changed over time. Articles with a high number of co-citations were considered to hold an authoritative position in the field and were able to suggest the necessary intellectual context for the study. 2016 Hua Zhang et al. published *Dietary polyphenols, oxidative stress, and antioxidant and anti-inflammatory effects* in *Current Opinion in Food Science* was the most cited article (n = 618), which pointed out that phenolic compounds, such as phenolic acids, flavonoids, and proanthocyanidins, have protective effects against biotic and abiotic stresses. It summarizes the research progress on dietary polyphenols, explores their antioxidant and anti-inflammatory activities, and investigates their involvement in inflammation-mediated metabolic disease mechanisms. Followed by the paper *The therapeutic potential of resveratrol: a review of clinical trials* (n = 211) by Adi Y Berman et al., published in the journal *NPJ precision oncology* in 2017. A citation burst, defined as a significant increase in the frequency of citations for a specific reference over a specific period, signifies the rapid identification and dissemination of that reference within the field of study. We display the top 25 citation surges (**Figure 7B**), with 16 articles receiving high citations over a period exceeding 3 years. During the period under investigation, a large number of widely cited articles in the field of RSV led to the explosion of cited literature, which began in 2014. Rossouw JE’s 2012 article in *Cell* entitled *Resveratrol ameliorates aging-related metabolic phenotypes by inhibiting cAMP phosphodiesterases* was the most explosive article (n = 75.98). The article asserts that red wine commonly contains RSV, a polyphenol believed to possess anti-aging and anti-diabetic properties. It increases NAD(+) and Sirt1 activity by competitively inhibiting cAMP-degrading phosphodiesterases, raising cAMP levels, increasing intracellular Ca(2+) levels, and activating the CamKKβ-AMPK pathway. In addition, the metabolic benefits of RSV, including the prevention of diet-induced obesity, improvement of mitochondrial function, physical strength, and glucose tolerance, can be mimicked by the PDE4 inhibitor rolipram. Thus, the application of phosphodiesterase 4 (PDE4) inhibitors may contribute to the prevention and amelioration of metabolic diseases associated with aging. In recent years, cited articles have focused more on RSV’s novel therapeutic modalities for various diseases and their molecular mechanisms, illustrating that RSV has demonstrated its positive effects on human health in several ways. The primary goal is to alleviate diseases in various tissues by increasing antioxidant capacity and modulating various signaling pathways. We discuss the application of RSV composite nanoparticles in drug delivery. Preventive and clinical measures for RSV are summarized to provide guidance for the correct understanding of RSV, accurate diagnosis, and effective prevention and treatment of RSV.

**Table 5:** Highly cited literature with top 10 studies in RSV.

Number	Title	Number of citations	Years of publication	Magazine	Author	Article type	Summary	DOI
**1**	Dietary polyphenols, oxidative stress and antioxidant and anti-inflammatory effects	618	2016	Current Opinion in Food Science	Hua Zhang	review paper	Phenolic compounds such as phenolic acids, flavonoids, and anthocyanins have a protective effect against biotic and abiotic stress. Fruits, vegetables, grains, spices, and herbs primarily contain these compounds, and a high intake of these compounds can reduce the risk of oxidative stress-related diseases. This paper aims to summarize the research progress of dietary polyphenols, explore their antioxidant, anti-inflammatory, and metabolic disease mechanisms involved in inflammation mediation, and look forward to future research directions.	(Zhang and Tsao, 2016)
**2**	The therapeutic potential of resveratrol: a review of clinical trials	211	2017	NPJ precision oncology	Adi Y Berman	review paper	RSV has anti-inflammatory and antioxidant effects and has an impact on the occurrence and development of many diseases. This paper reviews the clinical data of RSV and finds that it performs well in nervous system diseases, cardiovascular diseases, and diabetes but may have negative effects in some cancers and NAFLD, which provides useful guidance for the design of preclinical and clinical studies of RSV.	(Berman et al., 2017)
**3**	Novel insights of dietary polyphenols and obesity	137	2014	The Journal of Nutritional Biochemistry	Shu Wang	review paper	This paper investigated the role of dietary polyphenols in the prevention of obesity and obesity-related chronic diseases, with a special focus on green tea catechins, epigallocatechin gallate, RSV, and curcumin. Cellular studies revealed that these polyphenols reduce adipocyte activity, inhibit adipocyte differentiation and triglyceride accumulation, and reduce inflammatory responses. In addition, they also affect obesity by modulating various signaling pathways and biological processes such as adipogenesis, antioxidants, and anti-inflammatory responses.	(Wang et al., 2014)
**4**	Resveratrol: A Double-Edged Sword in Health Benefits	129	2018	Biomedicines	Bahare Salehi	review paper	This paper describes the structure of RSV, its origin, and its role in medicine. RSV, as a polyphenolic stilbene compound, is widely found in plants, especially grape skins and seeds, and in foods such as red wine. It has a strong antioxidant potential and antipathogenic capacity and is considered a plant antitoxin. In addition, RSV has shown a variety of biological activities, such as anti-tumor, anti-inflammatory, cardioprotective, vasorelaxant, phytoestrogenic, and neuroprotective. However, its application in the pharmaceutical field still faces challenges, including solubility, bioavailability, and adverse effects.	(Salehi et al., 2018)
**5**	Applications of nanoparticle systems in drug delivery technology	110	2018	Saudi Pharm J	Syed A A Rizvi	review paper	This paper explores the opportunities for nanoparticle-based drug formulation development in addressing and treating complex diseases. By modulating size, surface properties, and materials, nanoparticles can form smart systems that encapsulate therapeutic and imaging agents and materials. These systems can precisely deliver drugs to specific tissues and provide controlled-release therapies, thereby reducing drug toxicity and improving patient compliance. Nanotechnology has made significant advances in the treatment of diseases such as cancer and AIDS and has facilitated the development of diagnostic tests.	(Rizvi and Saleh, 2018)
**6**	The effects of polyphenols and other bioactives on human health	95	2019	Food & Function	César G Fraga	review paper	Different types of polyphenols may reduce the risk of various chronic diseases, e.g., flavan-3-ols in cocoa are associated with cardiovascular health, while the flavonoids quercetin and stilbene RSV are associated with cardiometabolic health. Polyphenols may also have clinical effects because they influence the gut microbiota and interact with other phytochemicals.	(Fraga et al., 2019)
**7**	Curcumin, the golden nutraceutical: multitargeting for multiple chronic diseases	79	2017	British Journal of Pharmacology	Ajaikumar B Kunnumakkara	review paper	Turmeric contains a pigment called curcumin. Numerous studies have demonstrated its antimicrobial and anti-inflammatory activity and its positive effects on a variety of chronic diseases, such as cancer, diabetes, and cardiovascular disease. It has anti-inflammatory properties because it inhibits various cell signaling pathways. In addition, curcumin has synergistic effects with other nutraceuticals.	(Kunnumakkara et al., 2017)
**8**	Autophagy and apoptosis dysfunction in neurodegenerative disorders	72	2014	Progress in Neurobiology	Saeid Ghavami	review paper	Autophagy and apoptosis are crucial physiological processes that maintain cellular homeostasis. Autophagy involves the removal of long-lived proteins and damaged organelles, while specific morphological features mediate apoptosis. Neurodegenerative diseases are believed to develop due to disruptions in autophagy. In this paper, we briefly review the recent advances in autophagy in neurodegenerative diseases and introduce the roles of autophagy and apoptosis in maintaining brain homeostasis. Finally, we discuss the potential applications of autophagy and apoptosis regulation in the treatment of neurodegenerative diseases.	(Ghavami et al., 2014)
**9**	Slowing ageing by design: the rise of NAD+ and sirtuin-activating compounds	68	2016	Nat Rev Mol Cell Biol	Michael S Bonkowski	review paper	SIRT1-7 is a family of NAD-dependent deacetylases with remarkable ability to prevent disease and reverse aging. Improvements in organ function, physical endurance, disease resistance, and longevity have been demonstrated by modifying or treating mice with sirtuin-activating compounds (STACs) or NAD precursors. Experiments suggest that STACs may be safe and effective in treating inflammatory and metabolic disorders in non-human primates and humans.	(Bonkowski and Sinclair, 2016)
**10**	Resveratrol Attenuates Trimethylamine-N-Oxide (TMAO)-Induced Atherosclerosis by Regulating TMAO Synthesis and Bile Acid Metabolism via Remodeling of the Gut Microbiota	53	2016	mBio	Ming-liang Chen	original research	RSV is a natural plant antitoxin with anti-atherosclerotic effects, but its mechanism of action is still unclear. The findings showed that RSV could lower TMAO levels and weaken AS caused by TMAO. It did this by changing the microbiota in the gut and stopping TMA production. RSV also increased the number of good bacteria and sped up the release of bile acids and the elimination of feces, which changed the production of bile acids in the liver. This process involves multiple biochemical pathways, including the regulation of the intestinal FXR-FGF15 axis.	(Chen et al., 2016)

### 3.7 Analysis of research keywords in the field of RSV

**Figure 8:**
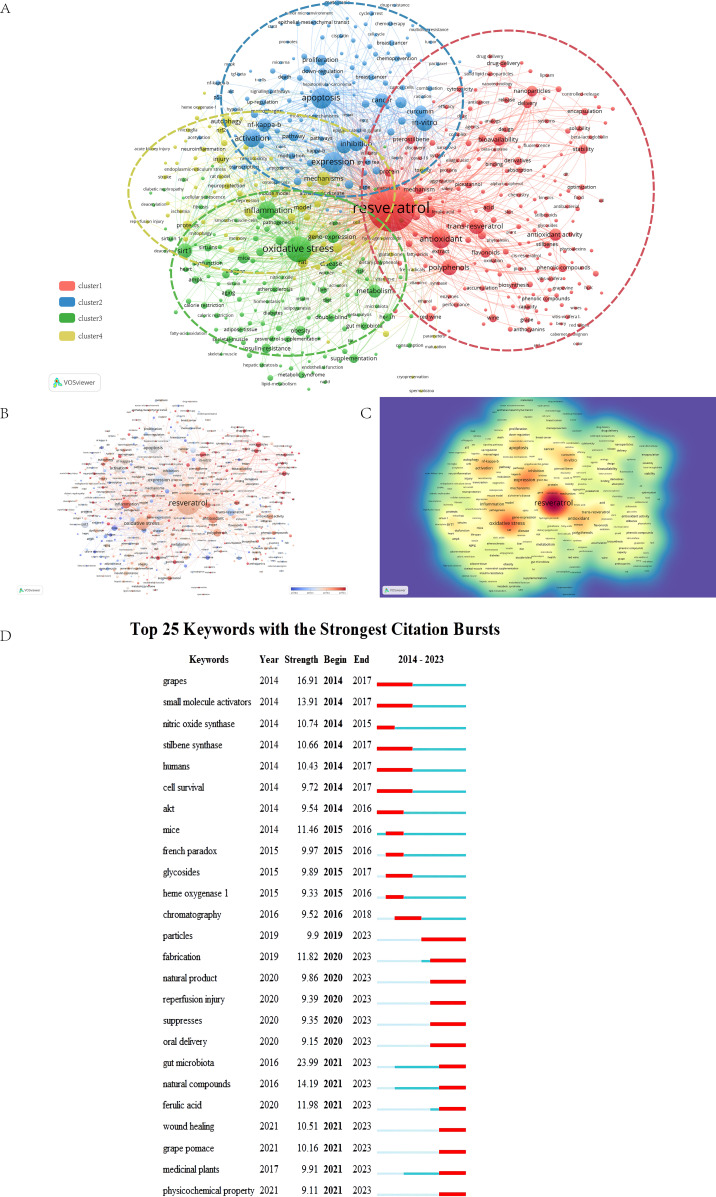
**(A)** Keyword clustering visualization The size of the circles is positively correlated with the frequency of keyword occurrences. The thickness of the connecting lines between the circles is related to the strength of the relationship between the keywords. Nodes of different colors form different clusters, and each color represents a different research direction. A co-occurrence clustering algorithm is performed on the keywords by calculating the keyword similarity to cluster the keywords with high similarity. **(B)** Keyword Strength Visualization Temporal Overlay. The size of the circle corresponds positively to the frequency of keyword occurrences. The average year of occurrence of each keyword is indicated by the gradient in the lower right corner. Blue indicates keywords with earlier occurrences, and red indicates more recent occurrences that may evolve into new research directions. **(C)** Keyword density visualization plot. The depth of color is proportional to the frequency of keyword occurrences. **(D)** The top 25 most frequently cited keywords in CiteSpace.

Keywords are summaries of the core content of an article and can be used to analyze the research hotspots and frontiers in the field of RSV. A total of 1000 keywords were obtained in this study.1 keyword had more than 10,000 occurrences, and 13 keywords had more than 1000 occurrences. Eight of them have a total link strength of more than 10000, and the rest of them are more than 1000. As a keyword included in the search formula, resveratrol (11038) occupies the No. 1 position in the number of occurrences of the keyword. Then VOSviewer was used to cluster the keywords as a basis for summarizing the research themes and hotspots, helping scholars to understand the direction and trend of the research focus in the field and clarifying the relationship between different themes in the field. The whole cluster analysis network is highly connected with a strong co-occurrence relationship, in which many node keywords, such as oxidative stress, apoptosis, expression, antioxidant, activation, *in vitro*, inflammation, etc., play an important role in connecting other branches of the field (**Figure 8A**). The red cluster emphasizes RSV’s capacity to function as a potent antioxidant, including antioxidants, trans-resveratrol, and polyphenols. RSV mitigates oxidative stress-induced damage to cells and tissues by neutralizing free radicals and bolstering the cells’ antioxidant defense mechanisms. The blue cluster, encompassing NF-kappa B, cancer, access, and apoptosis, highlights RSV’s potential in oncological therapies. Research in the green clusters—oxidative stress, inflammation, SIRT1, obesity, and diabetes—spotlights the anti-inflammatory and metabolic regulatory properties of RSV. Lastly, investigations within the yellow cluster—mechanics, injury, autophagy, and protection—concentrate on RSV’s roles in cellular protection and repair. Based on the keyword cluster analysis, we present the prominent keywords across different years, distinguished by various colors (**Figure 8B**). The keyword clusters that ignited the research surge in the early year (2014) were mice and red wine. Keywords such as grapes, small molecule activators, nitric oxide synthase, stilbene synthase, human cell survival, mice, French paradox, glycosides, heme oxygenase, and chromatography have recently diminished in relevance. In contrast, nanoparticles, drug delivery, and gut microbiota have seen a rise in popularity. We illustrate the research hotspots and developmental trends of RSV through the density of the keyword co-occurrence network (**Figure 8C**). By integrating these two figures, the intensity of the color corresponds to the frequency of occurrences. This allows us to discern the distribution patterns of each keyword within the field.Keywords with strong citation bursts can also reflect some emerging academic trends and hot topics in the field and can be used to predict the direction of cutting-edge research and reveal potential hot spots in the field. We list the top 25 most-cited keywords (**Figure 8D**). Gut microbiota exhibited the highest burst intensity related to RSV (intensity = 23.99), followed by grapes (n = 16.91). The RSV keyword burst is delineated into two distinct phases. In the first phase (2014-2017), prominent keywords included grapes, small molecule activators, nitric oxide synthase, stilbene synthase, humans and cell survival, with additional attention. The second phase (2020-2023) features keywords such as gut microbiota, natural compounds, femulic acid and wound healing, reflecting current research hotspots and emerging frontiers in the field.

## 4 Discussion

Commonly used analytical tools in the field of bibliometrics include CiteSpace, VOSviewer, and Bibliometrix in R. CiteSpace helps to discover research frontiers and hot topics by deeply analyzing citation relationships in the literature and displaying citation and collaboration vectors through network diagrams. VOSviewer generates scientific maps that show links between literature, authors, and journals, and provides impact assessment. Bibliometrix provides functions and tools for importing, processing, and cleansing literature data, including co-occurrence analysis, topic modeling, and collaborative network analysis, etc., with intuitive and easy-to-understand results. Together, these tools provide researchers with valuable information to gain a deeper understanding of the structure and trends of the discipline, to grasp research hotspots, and to assess the impact of research. RSV is a naturally occurring compound found in plants, particularly grapes, peanuts, and certain herbs. It is thought to have antioxidant and anti-inflammatory properties that may be beneficial in reducing the risk of heart disease and certain cancers. To date, no studies have been found on the bibliometric analysis of RSV. Although there are some studies supporting the benefits of RSV, more research is needed to confirm its effectiveness and safety. In addition, individual differences, intake doses, and long-term use may affect its effectiveness, so more in-depth knowledge is needed before recommending it for health purposes.

### 4.1 Global contribution to RSV research

Over the past decade, we have observed a continuous increase in academic publications in this research area. The significant increase in research activity in this field highlights its immense research value and may usher in new advances in the future. Papers were mainly published in Asian, North American, and European countries, with China having the highest number of publications related to economic development and the government’s emphasis on research. The United States follows China, with a strong collaborative relationship between the two countries. Nevertheless, the research on RSV is still insufficient, and international cooperation is particularly important to pool technology and resources to solve technical problems, promote the development of the RSV preparation process, and advance the field of Chinese medicine.

### 4.2 Research progress of RSV

In 1939, Japanese scientist Takaoka extracted RSV from the rhizome of the toxic plant *Veratrum grandiflorum* (Takaoka, 1939). RSV was first synthesized by Spath and Kromp using Perkin reaction in 1941 (Späth and Kromp, 1941). Studies on its antioxidant and health effects began in the 1980s, but did not attract widespread attention. In 1985, Moreno-Manas et al. used 3, 5-dihydroxytoluene as the starting material to prepare RSV through Wittig reaction, which had the disadvantages of low yield, low selectivity of ciso isomerism, and difficult to remove triphenyl phosphorus oxide as a by-product (Moreno and Pleixats, 1985). In the 1990s, researchers in Connecticut discovered RSV in wine (Jeandet et al., 1995). Sparked a global research boom. In 1997, RSV was confirmed to have anticancer activity (Jang et al., 1997). In 2003, Jeffery and Ferber successfully synthesized RSV by palladium-catalyzed one-pot method with a total yield of 80%. In recent years, this method has been widely concerned and has become the most promising method (Jeffery and Ferber, 2003). In 2003, *Nature* published a paper that RSV extended the life of yeast (Howitz et al., 2003), and in 2004 reported that RSV had a very clear effect on extending cell life (Wood et al., 2004). In 2006, its life-prolonging effect was demonstrated in mice (Baur et al., 2006). In 2008, *Cell Metabolism* revealed that RSV may affect the aging and health of the body (Pearson et al., 2008). After 2010, despite mixed research results and fraud scandals, RSV’s health benefits remain unproven. In 2012, *Cell* analyzed the molecular mechanism of RSV’s promotion of human health (Tennen et al., 2012). And RSV improved the metabolic phenotype associated with aging by inhibiting cAMP phosphodiesterase (Park et al., 2012). RSV is commercialized as a food and cosmetic ingredient and is sold as a food supplement. The current market is approximately $97.7 million and is expected to grow at a compound annual growth rate of 8.1% from 2018 to 2028 (Sáez-Sáez et al., 2020).

### 4.3 Research hotspots and frontiers of RSV

Keyword analysis plays a crucial role in the field of scientific research, not only reflecting the current research hotspots but also predicting the future development direction. From the latest keyword analysis data, we observe that the term “nanoparticle” has become the most popular research topic. This trend indicates that in future research, the combination of nanoparticles and RSVs will become a new trend. Topical application of nanomedicines can deliver therapeutic drugs directly into target tissues to exert therapeutic effects, and it is simple and easy to implement (Li et al., 2021b). Nano-formulated RSV effectively and significantly slowed the growth of colon cancer cells and the amount of hemoglobin in the tumor volume. It did this while remaining more bioavailable and effective (Sudha et al., 2020). When we combine nanoparticles and RSVs, we expect breakthroughs in drug discovery and disease treatment. The keyword “delivery” follows “nanoparticle.” In the field of drug delivery, nano-preparations such as nanoparticles and liposomes have demonstrated their importance in targeted therapy. Researchers have shown that solid lipid nanoparticles and nanostructured lipid carriers may be able to improve RSV’s low bioavailability and low ability to dissolve in water (Chimento et al., 2019). These nanoparticles have the unique advantage of improving drug stability and, in some cases, drug release (Kianfar, 2021). Studies have demonstrated that nanoparticles have been used to target RSV delivery to the brain, proving highly beneficial for treating neurological conditions such as cerebral hemorrhage (Mo et al., 2021). In particular, lipid nanoparticles, when functionalized with apolipoprotein E, can prevent the degradation of RSV in the bloodstream and achieve precise brain-targeted delivery (Neves et al., 2016). In addition, Wang et al. utilized a polylactic acid-hydroxyacetic acid copolymer to create inhalable RSV inclusion nanoparticles, which targeted drug concentration in the lungs and significantly enhanced RSV’s anticancer efficacy against non-small cell lung cancer (Qiu et al., 2020). Liposomes, as an innovative drug delivery system, offer numerous benefits including minimal adverse effects, high bioavailability, and effective targeting (Pandita et al., 2014). The RSV-loaded liposomes developed by Wiedenhoeft et al. were adept at delivering RSV to the cerebral microcirculation, mitigating oxidative stress in cerebral microvessels and thus safeguarding them (Wiedenhoeft et al., 2019). Nanocapsules have been found to be chemically multifunctional, which can not only improve the antioxidant activity and stability of drugs (Liu et al., 2020), but also deliver drugs in a targeted manner (Amin et al., 2018). RSV-loaded nanocapsules can traverse the blood-brain barrier, elevating RSV levels in brain tissue and providing neuroprotective effects in AD rats (Frozza et al., 2013). Moreover, encapsulating RSV in filipin protein nanoparticles allows for controlled drug release, enhancing drug stability and preventing unnecessary metabolic degradation, thus improving RSV’s ability to promote wound healing and extend its therapeutic duration (Lozano-Pérez et al., 2017).

The application of RSV in topical therapy is limited due to its low bioavailability and poor water solubility when taken orally (Valachová and Šoltés, 2021). Natural multicapsular compounds such as gellan gum, gelatin and pectin can form stable networks in acidic environments, but gellan gum is susceptible to high temperatures and has poor water solubility (Prezotti et al., 2020). The addition of RSV can reduce the attraction between carboxyl groups, thus reducing the swelling degree of the hydrogel and promoting the release of RSV (Wang et al., 2021). The combination of oils and polysaccharides formed heterogeneous hydrogels, which prolonged the active time of the drug in the blood and improved stability and bioavailability compared to free RSV (Joseph et al., 2022). High molecular weight chitosan protects RSV from UV degradation through the formation of nanogels and is further encapsulated in nuclear endosomes for improved targeting after cellular internalization (Buosi et al., 2020). Despite the poor mechanical strength of chitosan hydrogels, hydrogels constructed using bioactives have received attention in recent years, and the porous nanostructures formed by peptide moieties not only have good mechanical strength, but also provide a natural bioenvironment for RSV to exhibit excellent slow-release properties (Zhao et al., 2020).

In 2023, gut microbiota, natural compounds, wound healing, and other keywords will still maintain a strong explosive trend. The gut microbiota is increasingly recognized as a promising target for the treatment and prevention of inflammatory and metabolic disorders in humans (Bastos and Rangel, 2022). As a low molecular weight stilbene compound, RSV is predominantly metabolized in the large intestine, where it influences the gut microbiota. During its metabolism, RSV exits the gut through transporter proteins such as breast cancer resistance proteins and enters systemic circulation via multidrug resistance protein 2 (MRP2) on the apical membrane and MRP3 on the basolateral membrane (Abdelhafiz et al., 2023). Approximately 70-75% of RSV undergoes metabolism in the gut, with only 1-8% entering the bloodstream (Vesely et al., 2021). The villi play a crucial role in nutrient absorption and transport. Research indicates that RSV supplementation can enhance intestinal morphology (Qiu et al., 2021), including increasing jejunal villus height and length in mice with non-alcoholic fatty liver disease (NAFLD) (Wang et al., 2020a). Alterations in the composition of the intestinal microbiota have been linked to the onset and progression of numerous diseases, encompassing a variety of chronic conditions (Cai et al., 2020).

In the colon, the intestinal microbiota can further metabolize RSV and its metabolites, with dihydroresveratrol being the most common metabolite (Springer and Moco, 2019). The RSV treatment was able to raise the numbers of good bacteria, like Bifidobacteria and Lactobacilli, and lower the numbers of bad bacteria, like *E. coli* and Enterobacteriaceae (Larrosa et al., 2009). Researchers also found that RSV improved renal function, improved glucose homeostasis, restored intestinal permeability, and reduced the drive for inflammatory markers (Cai et al., 2020). Specifically, RSV significantly inhibited gut microbiota abnormalities induced by a high-fat diet, increased the growth of beneficial bacteria, and enhanced the diversity of gut microbiota, thereby preventing intestinal dysbiosis (Yi et al., 2014).

### 4.4 Clinical application of RSV

We discovered that terms with significant persistence include “drug delivery,” “antioxidant,” and “inflammatory.” Not only does the research in these domains have a strong basis, but it also continues to be valuable and interesting. In particular, research on anti-inflammatory and anti-oxidative stress is important in revealing disease mechanisms, developing novel drugs, and so on. RSV can down-regulate the expression of the inflammatory factors IL-1β, IL-6, IL-8, and TNF-α in a dose-dependent manner (Li et al., 2021c). In addition, RSV possesses strong antioxidant activity and can prevent cellular oxidative stress by targeting and regulating the PI3K/AKT and Wnt/β-catenin signaling pathways, increasing antioxidant content, and decreasing the production of ROS and the marker of oxidative stress, malondialdehyde (MDA) (Bahar and Singhrao, 2021; Li et al., 2021c).RSV prevents oxidative stress in cells by inhibiting signal transduction, signal transducer, and activator of transcription 1 (STAT1) (Li et al., 2021a), and activating the SIRT1/NF-κB signaling pathway (Yang et al., 2021a; Zhao et al., 2021)to reduce cellular inflammation and oxidative stress. According to Wang et al (Wang et al., 2020b), RSV reduced neuropathic pain by controlling the expression of triggering receptors on myeloid cells. This stopped microglia from causing neuroinflammation.

RSV features a 4′-hydroxyl group and exhibits intrinsic biocompatibility and non-cytotoxicity (Ma et al., 2021). Currently, the research hotspots for RSV mainly focus on its pharmacological effects, synthesis methods, and molecular mechanisms. The compound possesses anti-tumor, anti-inflammatory, antibacterial, and neuroprotective characteristics, among others, and a thorough study of its mechanisms of action and targets is necessary. Its extraction, purification, and preparation processes are also hot spots for research. Current research focuses on investigating the molecular mechanism of RSV’s pharmacological effects, particularly its anti-inflammatory and antioxidant mechanisms. Further investigation into the pharmacological and toxicological properties of RSV is necessary, as these aspects play a crucial role in the development of new drugs, thereby bolstering their clinical application. We should further study RSV, a common component in many traditional Chinese medicines, and explore its pharmacological mechanism of action to gain a deeper understanding of its role in traditional Chinese medicine therapy.

RSV has inhibitory effects on a wide range of cancer cells, while at the same time, it has no toxicity or side effects on normal cells. Novel targets and pathways continue to be identified. Wu et al. demonstrated that RSV primarily inhibits the proliferation of breast cancer cells by interfering with the cell cycle and inducing apoptosis (Wu et al., 2019). RSV inhibits the enhancer of zeste 2 (EZH2) through dephosphorylation of extracellular regulated protein kinases (ERK1/2), thereby curbing the proliferation and growth of breast cancer cells (Hu et al., 2019). Furthermore, RSV regulates the E2/ERα/NGB signaling pathway (Cipolletti et al., 2019), microRNAs (Zhang et al., 2019), and the Notch pathway to inhibit breast cancer cell proliferation. RSV also modulates the expression of programmed cell death-ligand 1 (PD-L1) via the Wnt pathway, where Sirtuin 1 (SIRT1) deacetylates and stabilizes transcription factors, inhibiting Axin2 transcription and promoting T cell factor (TCF) binding to the PD-L1 promoter (Yang et al., 2021b). In RSV-treated HCT116 cells, transforming growth factor beta-3 (TGF-β3) and tumor necrosis factor beta (TNF-β) expressions are notably reduced, alongside down-regulation of p65-NF-κB (Buhrmann et al., 2020). Buhrmann et al. were the first to show that TNF-β/TNF-β-receptor signaling regulates rectal cancer cell proliferation (Buhrmann et al., 2019b), with RSV down-regulating this inflammatory response and modulating NF-κB and focal adhesion kinase (FAK) to control rectal tumor cell growth (Buhrmann et al., 2019a), proliferation, and invasion. RSV and leucotaxol elevate PD-L1 expression in rectal cancer through NF-κB signaling mediated by histone deacetylase 3 (HDAC3)/p300, enhancing rectal cancer cell apoptosis (Lucas et al., 2018). RSV not only independently inhibits cancer cells but also augments the effects of drugs like rapamycin (Bian et al., 2020) and retinoic acid (Li et al., 2018). When combined with rapamycin, RSV inhibits tumor growth by modulating the PI3K/AKT/mTOR signaling pathway (Bian et al., 2020). Additionally, RSV decreases galectin-3 (GAL-3) levels by raising miR-424-3p, thereby inhibiting glycolysis and targeting the AMPK/mTOR signaling pathway to suppress ovarian cancer cell proliferation and induce apoptosis (El-Kott et al., 2019). Hao et al. found that RSV might reduce inflammation through the AMPK/Drp1 signaling pathway, thus alleviating bone cancer pain in rats (Hao et al., 2020).

As a natural phytoestrogenic agent, RSV acts as an estrogen receptor (ER) agonist, up-regulating vitamin D receptor expression in osteoblasts and promoting bone formation. In postmenopausal women, regular RSV supplementation significantly reduces CTX levels, enhances lumbar spine and femoral neck bone mineral density (BMD), and improves bone mineralization, with increased osteocalcin levels mitigating bone loss (Wong et al., 2020). RSV also shows potential as a therapeutic agent for neurodegenerative diseases such as Alzheimer’s and Parkinson’s diseases. Research indicates that RSV may exert neuroprotective effects in Alzheimer’s disease models through Sirt1 signal transduction (Ma et al., 2019). Kong et al. revealed that RSV enhances antioxidant capacity and estrogen levels in Alzheimer’s disease models via the Nrf2/heme oxygenase-1 (HO-1) signaling pathway (Kong et al., 2019).

Furthermore, RSV exhibits antibacterial and antiviral properties. Huang et al. discovered that RSV significantly down-regulates MEK1/2 and ERK phosphorylation in rotavirus-infected mouse models, alleviates diarrhea, and markedly reduces the mRNA expression levels of IL-2, IL-10, TNF-α, interferon-γ, macrophage inflammatory protein 1, and monocyte chemoattractant protein 1 in intestinal tissues, demonstrating its antiviral activity (Huang et al., 2020).

### 4.5 Research on drug development of RSV

In mammalian experimental models, RSV undergoes extensive metabolism and rapid elimination, leading to limited bioavailability. Following oral administration, RSV is absorbed at the intestinal level through passive diffusion or membrane transport proteins and subsequently released into the bloodstream, where it can be identified either as an unmodified compound or a metabolite (Varoni et al., 2016; Chimento et al., 2019). Despite 75% of RSV being absorbed orally, only 1% is detected in plasma after complete metabolism (Walle, 2011). Although RSV has the disadvantages of low bioavailability, poor water solubility, and rapid hepatic and intestinal metabolism, these problems can be solved or ameliorated by improved co-solvents and the use of nanoparticles, etc (Rizvi and Saleh, 2018). RSV can be absorbed in large quantities by intestinal cells by passive diffusion or carrier-mediated translocation across the apical membrane of the cell after oral administration and is then metabolized rapidly and extensively to RSV glucuronides or sulfate salts (Yu et al., 2002). Studies have shown that a large proportion of ingested RSV (approximately 90%) reaches the colon in an intact form and undergoes subsequent intestinal fermentation. When absorbed through the portal vein, the resulting polyphenol metabolites will enter the liver for further methylation, glucuronidation, or reaction with sulfate. Subsequently, the metabolites penetrate the circulation and reach the target tissues and cells to exert their biological activity. The bile can recycle excess RSV and its metabolites back to the small intestine, while the urine can excrete them (Springer and Moco, 2019). Recently, various methodological strategies and synthetic derivatives have been developed to improve RSV’s bioavailability. Numerous studies have focused on synthesizing novel and more potent RSV analogs with enhanced pharmacokinetic properties, reduced toxicity, and minimal side effects. Methoxylated, hydroxylated, and halogenated RSV derivatives have been particularly investigated due to their advantageous biological activities and improved oral bioavailability (Nawaz et al., 2017). Prior research indicates that methoxylation enhances metabolic stability and prolongs the duration required for the molecule to attain peak plasma concentrations (Ferraz da Costa et al., 2020).

In terms of enhancing wound healing, RSV fosters angiogenesis and suppresses inflammation (Huang et al., 2019; Zhao et al., 2020). However, its hydrophobic nature results in limited *in vivo* delivery efficiency (Jeandet et al., 2021). To address this, a variety of advanced wound dressings have been developed, such as hydrogels (Yang et al., 2022) and biological scaffolds (Poornima and Korrapati, 2017), which effectively preserve RSV’s biological activity and facilitate integration with adjacent tissues. Researchers have created an electrospun scaffold incorporating RSV using polycaprolactone (PCL) and a chloroform-dimethylformamide solvent mixture, which accelerates wound closure and re-epithelialization (Lakshmanan et al., 2019). The subsequent application of a double-layer stent combined with hydrogel has further augmented hemostasis and reduced swelling (Ma et al., 2021). Additionally, studies indicate that integrating RSV with bacterial cellulose promotes re-epithelialization of rat epidermis (Meng et al., 2019). Furthermore, RSV-loaded nanovesicles combined with wafers extend the drug’s residence time in the skin (Amanat et al., 2020). Vesicles containing RSV and gallic acid exhibit potent antioxidant and antibacterial properties, effectively shielding the skin from environmental pollutants and damage (Vitonyte et al., 2017).

Researchers conducted pharmacokinetic studies to investigate the pharmacokinetic properties of RSV *in vivo*, including absorption, distribution, metabolism, and excretion, to provide theoretical support for its rational use. In the meantime, researchers can explore drug interactions, including their synergistic and antagonistic effects, to guide the rational combination of clinical medications. Future studies can establish quality control methods for RSV, such as fingerprinting, content determination, and purity testing, to ensure the quality and safety of Chinese medicine products. In the meantime, researchers can develop novel pharmaceutical formulations of RSV, such as oral, injection, and topical formulations, to enhance the bioavailability and efficacy of RSV. Researchers have discovered various types of RSV formulations and carriers. Many new things have been made, such as RSV self-microemulsifying tablets (Bolko Seljak et al., 2018), trans-RSV nanohybrids (Singh et al., 2017), microcapsules (Luzardo-Álvarez et al., 2019), poly (-caprolactone) microcapsules, and RSV liposomes (Wiedenhoeft et al., 2019). These formulations and carriers were able to improve the solubility, stability, bioavailability, and antioxidant and anti-inflammatory properties of RSV.

## 5 Conclusion

The citation analysis was performed using CiteSpace software, which generated a network diagram showing the research landscape in a visual way. The graph details citation relationships, co-authorships, and other relevant metrics between different publications, thus providing insight into the structure and trends of the research landscape. This study focuses on analyzing the literature related to RSV in the WoSCC between 2014 and 2023. In addition to CiteSpace, software such as VOSviewer was used to quantitatively visualize the number of publications, authors, countries, and research institutions. This study analyzes in detail the development of RSV research. According to the study, the number of RSV-related articles on WoSCC has shown a continuous growth trend, with an average of about 1693.4 articles being published each year, and the overall trend has been on the rise. In particular, China has published the largest number of papers in this field of research, reaching 5,877, while China Medical University is one of the institutions with the highest number of published papers and citations in this field. In addition, the research team is mainly led by Prof. Richard Tristan. The most published papers were *Molecules*, which is an academic journal of chemistry-organic chemistry, focusing on the frontiers of materials chemistry. *Dietary polyphenols, oxidative stress, and antioxidant and anti-inflammatory effects* is the most frequently cited article (Zhang and Tsao, 2016). Keywords such as oxidative stress, apoptosis, and gene expression play a key role in connecting the field to other branches. These results highlight hot spots and important directions in the field of RSV research. Future research challenges include how to alter the structure of RSV to obtain derivatives with higher bioactivity and bioavailability. The number of people focusing on RSV research is expected to increase with the rise of emerging disciplines and specialties. Research on nanoparticles, drug delivery, and the gut microbiota is also becoming of increasing interest.

Future research should focus extensively on the pharmacological effects of RSV, including antioxidant, anti-inflammatory, and anticancer aspects, as well as its specific components and molecular mechanisms. With the help of currently available research results, drugs can be optimized by means of nanomaterial drug delivery to improve their efficacy, and increase their bioavailability. These studies support subsequent *in vitro* and *in vivo* preclinical studies, laying a solid foundation for drug development and clinical translation. In-depth studies of the pharmacology, toxicology, and pharmacokinetics of RSVs will provide a better understanding of their mechanism of action and support future medical advances.

## 6 Strengths and limitations

The field of RSV research is rapidly developing, with a variety of research hotspots and a promising future. These analyses aimed to explore the international distribution pattern of RSV research, collaborative relationships among researchers, and major research directions in the field. This study will help advance the field of RSV and provide direction and lessons for future research. This work uses the WoSCC as its primary data source and rigorously restricts the screening to English literature following a careful review and search of the literature. This change entails the disregard of non-SCI publications and literature published in languages other than English, even though its goal is to concentrate more intently on excellent research published in SCI-indexed journals. While CiteSpace and VOSviewer are valuable visualization tools for literature combing, they are not a perfect substitute for a methodical literature search procedure. This could add some material that isn’t very relevant to the subject throughout the literature screening procedure, which could skew the conclusions of the analysis as a whole. However, given that these effects are relatively small, they are unlikely to change the main trends and characteristics revealed by the research subject.

This paper restricts the literature inclusion timeline to the end of 2023 in order to capture the immediate dynamics of research hotspots and frontiers. However, it is worth noting that due to the relatively short publication period of the literature in 2023, sufficient citation data has not yet been accumulated, which may weaken the persuasiveness of our findings to some extent. As such, this factor must be properly taken into account when interpreting the analysis’s findings. Despite these drawbacks, the study offers a clear picture of the state of RSV today and in the future.

## Data Availability

The original contributions presented in the study are included in the article/supplementary material. Further inquiries can be directed to the corresponding author.
